# Detection of Periodontal Pathogens from Dental Plaques of Dogs with and without Periodontal Disease

**DOI:** 10.3390/pathogens11040480

**Published:** 2022-04-17

**Authors:** Jana Kačírová, Miriam Sondorová, Aladár Maďari, Eva Styková, Rastislav Mucha, Radomíra Nemcová, Nikola Marečáková, Jana Farbáková, Marián Maďar

**Affiliations:** 1 Department of Microbiology and Immunology, University of Veterinary Medicine and Pharmacy in Kosice, Komenskeho 73, 041 81 Kosice, Slovakia; kacirova.jana@gmail.com (J.K.); miriam.sondorova@gmail.com (M.S.); radomira.nemcova@uvlf.sk (R.N.); 2 Small Animal Clinic, University Veterinary Hospital, University of Veterinary Medicine and Pharmacy in Kosice, Komenskeho 73, 041 81 Kosice, Slovakia; aladar.madari@uvlf.sk (A.M.); n.marecakova@gmail.com (N.M.); jana.farbakova@uvlf.sk (J.F.); 3 Clinic of Horses, University Veterinary Hospital, University of Veterinary Medicine and Pharmacy in Kosice, Komenskeho 73, 041 81 Kosice, Slovakia; eva_stykova@yahoo.com; 4 Institute of Neurobiology, Biomedical Research Center of the Slovak Academy of Sciences, Soltesovej 4, 040 01 Kosice, Slovakia; rastislavmucha@gmail.com

**Keywords:** dog, periodontal disease, *Porphyromonas gulae*, *Tannerella forsythia*, *Treponema denticola*, *Treponema putidum*, PCR

## Abstract

Dental plaque bacteria are one of the main factors responsible for the development of a periodontal disease, which is the most common infectious disease in dogs. The aim of this study was to identify the presence of periodontal disease-related bacteria in the dental plaque of dogs. Plaque samples were taken from dogs with and without periodontal disease. Samples were analyzed for the presence of *Porphyromonas gulae*, *Tannerella forsythia* and *Treponema denticola* using a PCR technique amplifying 16S rRNA genes of *P. gulae* and *T. forsythia* and *flaB2* genes of *Treponema* species, including *T. denticola*. The presence of *T. forsythia* was confirmed in all samples. *P. gulae* was detected in all dogs with periodontal disease and in 71.43% of dogs without periodontal disease. *Treponema* spp. were detected in 64.29% of the samples. Based on Sanger sequencing and Basic Local Alignment Search Tool algorithm, *Treponema* spp. were identified as *T. denticola* and *Treponema putidum*. *T. denticola* was present in 28.57% of dogs with periodontal disease, while *T. putidum* was present in 42.86% of dogs with periodontal disease and in 57.14% of dogs without periodontal disease. *T. putidum* was positively correlated with both *P. gulae* and *T. forsythia*, suggesting that it may be involved in the development of periodontal disease.

## 1. Introduction

In veterinary medicine, periodontal diseases are the most common diseases of domestic carnivores and occur in approximately 80% of dogs older than 2 years [1,2]. The incidence of the disease increases significantly with age and weight of the animals. Epidemiological studies indicate higher occurrence in small breeds [3,4]. Based on the clinical signs, periodontal diseases are usually divided into two groups, gingivitis and periodontitis. The most common clinical signs of periodontal disease include halitosis, ptyalism, altered gingival color, gingival bleeding, tooth mobility, anorexia and behavioral changes. Periodontal diseases are the result of the accumulation of dental bacterial plaque on the surface of the teeth, which is exacerbated by the accumulation of mineralized dental calculus [5,6]. 

**Figure 1:**
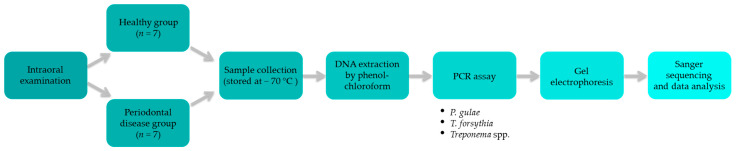
Flowchart of sample processing.

Periodontitis is a dysbiotic disease rather than an infection caused by only a few selected species of microorganisms. Dysbiosis of the periodontal microbiota represents a change in the relative number of individual components of the bacterial community, namely, the shift of subgingival Gram-positive bacteria to Gram-negative bacteria [7,8]. *Porphyromonas gingivalis*, *Treponema denticola* and *Tannerella forsythia* (formerly *Bacteroides forsythus*) are considered to be the most important pathogens in periodontal disease in humans [9]. *Porphyromonas gulae*, previously known as the animal biotype of the human periodontal pathogen *P. gingivalis*, has virulence properties that are associated with *P. gingivalis* and may play a key role in the pathology of periodontitis in companion animals [10]. Several different species of *Treponema* are found in the oral cavity of healthy dogs, which suggests that they are a part of normal microbiota [11]. However, *T. denticola* is most commonly associated with periodontal disease [12,13]. The meaning of *Treponema* spp. in the etiology of periodontal disease is still not entirely clear and requires further investigation [11]. The presence of *T. forsythia* in human dental plaque is associated with an increased risk of periodontitis [14], while in dogs, studies show conflicting results in the presence of *T. forsythia* in dental plaque [15,16]. The aim of present study was to determine the presence of *P. gulae*, *T. denticola* and *T. forsythia* in supragingival dental plaque samples of dogs with and without periodontal disease by PCR (Figure 1).

## 2. Results

### 2.1. Study Population

Nine female and five male dogs of different breeds aged between 1–14 years were sampled. Based on the clinical signs, such as the amount of plaque, presence of dental calculus, inflammation of the gums or tooth loss, they were divided into two groups—healthy and periodontal disease (Table 1).

### 2.2. Molecular Analysis

**Figure 2:**
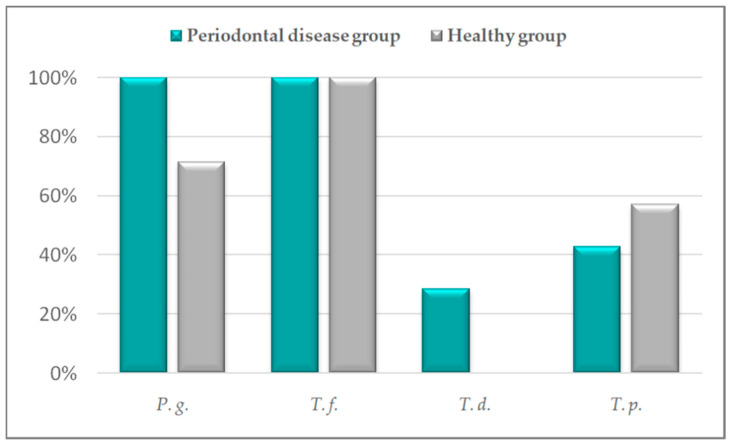
Detection of periodontal pathogens in dental plaques of healthy and periodontal disease groups of dogs. *P. g.*—*Porphyromonas gulae*, *T. f.*—*Tannerella forsythia*, *T. d.*—*Treponema denticola*, and *T. p.*—*Treponema putidum*.

Species-specific PCR using the primers targeting species *P. gulae* showed that *P. gulae* was detected in all dogs with periodontal disease and in 71.43% of healthy dogs. Using specific PCR for *Treponema* species, including *T. denticola*, the presence of *Treponema* spp. was detected in 71.43% of dogs with periodontal disease and in 57.14% dogs without periodontal disease. *T. forsythia* was detected in all dogs with and without periodontal disease by species-specific PCR. The presence of *P. gulae* and *T. forsythia* was confirmed by Sanger sequencing and Basic Local Alignment Search Tool algorithm. Based on these methods, *Treponema* spp. were identified as *T. denticola* and *Treponema putidum*. *T. denticola* was present in 28.57% of dogs with periodontal disease, *T. putidum* in 42.86% of dogs with periodontal disease and in 57.14% of healthy dogs. The results of detected periodontal pathogens in samples of dental plaques of healthy and periodontal disease groups are shown in Figure 2.

*T. denticola* was not present in any healthy dog. All three pathogens, *P. gulae*, *T. forsythia* and *T. denticola*, were detected in only two dogs with periodontal disease (28.57%). *P. gulae* together with *T. forsythia* were present in all dogs with periodontal disease and in five healthy dogs (71.43%) (Table 2).

## 3. Discussion

Periodontal diseases represent a serious diagnostic and therapeutic problem in human and veterinary medicine. Therapy of periodontal diseases is focused on suppressing the progression of inflammation and often involves a combination of different therapeutic approaches, such as scaling and root planing, the use of antibiotics and antimicrobial agents (e.g., chlorhexidine), antimicrobial photodynamic therapy or even the use of probiotics [17,18]. Moreover, dental chews can be used in dogs to reduce periodontal disease by beneficially shifting the microbiota of dental plaque [19]. Periodontal pathogens from the dental plaque, apart from irreversible damaging periodontium, can also cause some systemic diseases, which points to the importance of their identification [16].

Both in humans and animals, composition of bacterial plaque has been studied for many decades using culture methods. In recent times, methods of molecular biology have come to the foreground because of its high specificity, low time-consuming character and technical demand. In the present study, we used molecular methods for the detection of *P. gulae*, *T. forsythia* and *T. denticola*, which are associated with canine periodontal disease. However, various research studies reported their different prevalence in plaque samples of dogs.

Özavci et al. (2019) detected the presence of *P. gulae* in dogs with periodontal disease in only 39% of samples [20]. Although, in the study of Senhorinho et al. (2011), the presence of *P. gulae* was observed in 56% of dogs without periodontitis and 92% of dogs with periodontitis [21]. Kato et al. (2011) reported the presence of *P. gulae* in 92.31% of dogs [15] and Yamasaki et al. (2012) in 71.2% of dogs [22]. In the present study, *P. gulae* was detected in 100% of dogs with and in 71.43% of dogs without periodontal disease.

Gołyńska et al. (2017) found *T. forsythia* in only one female dog, and in male dogs, they did not isolate this bacterium at all [16]. Özavci et al. (2019) detected the presence of *T. forsythia* in 4% of dogs with periodontal disease [20], which contradicts the results of other studies and the present study. Di Bello et al. (2014) identified *T. forsythia* in 67.12% of dogs [23] and Yamasaki et al. (2012) in 77.3% of dogs [22]. Kato et al. (2011) detected *T. forsythia* in almost all dogs analyzed; therefore, they consider it a common member of the canine oral microbiota [15]. In the present study, all dogs with and without periodontal disease were positive for *T. forsythia*. 

Taxa belonging to the genus *Treponema* are common members of the microbial community of the human oral cavity. However, specific treponemes may be involved in the etiopathology of periodontal disease [24]. Valdez et al. (2000) confirmed the presence of *T. denticola*, *T. socranskii* ssp., *T. vincentii*, *T. maltophilum*, *T. medium* and *T. pectinovorum* in dental biofilm of dogs [25]. In the present study, consensus primers for the *flaB2* gene for the detection of *T. denticola*, *T. vincentii*, *T. medium* ssp. *bovis* and *T. phagedenis* ssp. *vaccae* were used. The *flaB2* gene has been previously used to determine *Treponema* species associated with bovine digital dermatitis [26,27].

Several studies report a prevalence of *T. denticola* in dogs of less than 7% [15,20,22,23]. In addition, Nishiyama et al. (2007) did not detect *T. denticola* in samples from dogs with periodontitis [28]. On the other hand, Gołyńska et al. (2017) observed *T. denticola* in all tested dogs [16]. In the present study, *T. denticola* was detected in 28.57% of dogs with periodontal disease but not in dogs without periodontal disease. Additionally, the presence of *T. putidum* was determined in the plaque samples taken from dogs with and without periodontal disease. 

Oral treponemes are classified into 10 phylogroups. *T. denticola* and *T. putidum* belong to phylogroup 2 and share 98.5% of their 16S rRNA gene sequence homology [29]. Both *T. putidum* and *T. denticola* are associated with human periodontal disease. *T. putidum* was isolated for the first time from human periodontitis lesions and acute necrotizing ulcerative gingivitis sites [30]. *T. putidum* has homologues of virulence factors previously described within *T. denticola*, such as factor H binding protein implicated in evading complement-mediated killing, the major surface protein involved in cellular adhesion processes and cystalysin (hemolysin) involved in volatile sulfur compound production and erythrolysis [29]. In the present study, the presence of *T. putidum* was in positive correlation with both *P. gulae* and *T. forsythia*. Nises et al. (2018) detected *T. putidum* in samples from dogs with periodontal disease [11]. Based on these findings, we can assume that *T. putidum* also plays a role in the development of canine periodontal disease.

## 4. Materials and Methods

### 4.1. Animals and Sampling

A total of 14 dogs of different breeds, age and periodontal status were sampled at the Small Animal Clinic, University of Veterinary Medicine and Pharmacy in Kosice. Informed consent was obtained from the owners of the dogs for the study. The study was approved by the Ethics Commission of the University of Veterinary Medicine and Pharmacy in Kosice. Prior to the collection of dental plaque, an intraoral examination was performed to assess the periodontal status of each non-anesthetized dog by a veterinarian. The stage of periodontal disease was assessed according to Bauer et al. (2018) [31]. The dental plaque samples were taken from the buccal surfaces of the right upper canines and premolars with a syringe needle into an Eppendorf tube containing 300 µL of phosphate-buffered saline. After sampling, samples were stored at −70 °C until DNA extraction and PCR analysis were performed. 

### 4.2. DNA Extraction 

Thawed samples were centrifuged at 10,000× *g* for 10 min at 4 °C and the supernatant was discarded. The protocol for DNA extraction according to Vesty et al. (2017) [32] with some modifications was performed. Briefly, the pellet was resuspended in 180 µL of 10% sodium dodecyl sulphate and 25 µL of proteinase K was added to the mixture. The tubes were incubated at 55 °C for 2 h, with shaking at 300 rpm. Proteinase K was inactivated by heating at 95 °C for 5 min [33]. The tubes were then centrifuged at 10,000× *g* for 5 min at 23 °C and the supernatant was transferred to the Eppendorf tubes. Phenol and chloroform were equally (1:1) added to the supernatant and centrifuged at 10,000× *g* for 5 min at 23 °C. The upper aqueous phase was transferred to the Eppendorf tube. Isopropanol (0.6 volume of supernatant) and 3 M sodium acetate solution (0.1 volume of supernatant) were added. The nucleic acids were precipitated overnight at 4 °C. The following day, DNA was pelleted at 10,000× g for 10 min at 4 °C, washed with 100 µL of cold 70% ethanol and dried at 35 °C for 10 min. The pellets were resuspended in 30 μL of TE buffer [50 mM Tris-HCl, 10 mM EDTA]. The concentration of DNA was measured (NanoDrop 1000, Thermo Fisher Scientific, Waltham, WA, USA) and samples were diluted to a concentration of 50 ng/µL of template DNA. 

### 4.3. PCR Assay

The PCR was processed using 2 µL (100 ng) of the sample (DNA template) added to 50 μL of reaction mixture containing OneTaq 2× Master Mix with Standard Buffer (New England Biolabs, Foster City, CA, USA), molecular grade water and primers. The primers used for PCR amplification of *P. gulae*, *T. forsythia* and *Treponema* species, including *T. denticola*, and PCR cycling conditions are listed in Table 3. PCR amplifications were performed in a thermocycler (TProfessional Basic, Biometra GmbH, Göttingen, Germany). A negative control (RNAse free H_2_O) was included in each PCR run. The amplicons were separated by gel electrophoresis and visualized with GelRed (Biotium, Inc., Hayward, CA, USA) under UV light [34]. A 100 bp DNA ladder (New England Biolabs, Foster City, CA, USA) was used as a molecular size standard.

### 4.4. Sequencing and Data Analysis

The amplification products were sent for Sanger sequencing in both forward and reverse directions (Microsynth, Wien, Austria). The obtained chromatograms of sequences were edited and aligned using Geneious alignment in Geneious 8.0.5 (Biomatters, Auckland, New Zealand). Homology searches were performed using the Basic Local Alignment Search Tool (BLAST) algorithm at the National Center for Biotechnology Information (NCBI). The nucleotide sequences were deposited in GenBank with accession numbers from MW595983 to MW595989, MW604827, MW604828, MW604829, MZ215849 and MZ215850, from OL839920 to OL839933, from OL906423 to OL906426, and from OM196210 to OM196214. 

## 5. Conclusions

Based on our results, it can be assumed that *T. forsythia* is a common member of the oral microbiota of dogs, whereas *T. denticola* was detected only in dogs with periodontal disease. The higher prevalence of *P. gulae* was observed in dogs suffering with periodontal disease. Moreover, *T. putidum* may also be involved in the development of this disease. Further investigation is needed to clarify the possible co-involvement of *T. putidum* in the development of canine periodontal disease, and a larger sample size of studied population of dogs is needed as well.

## Figures and Tables

**Table 1:** General information and periodontal status of the sampling dogs.

Dog	Breed	Age (Years)	Sex	Periodontal Status
1	Jack Russell Terrier	14	♂	Periodontal disease
2	Yorkshire Terrier	6	♀	Periodontal disease
3	Maltese	8	♀	Periodontal disease
4	Maltese	3	♀	Periodontal disease
5	Prague Ratter	11	♂	Periodontal disease
6	Chihuahua	9	♂	Periodontal disease
7	Labrador Retriever	10	♀	Periodontal disease
8	German shepherd	1	♀	Healthy
9	German shepherd	1	♀	Healthy
10	German shepherd	1	♀	Healthy
11	German shepherd	1	♀	Healthy
12	German shepherd	1	♀	Healthy
13	German shepherd	1	♂	Healthy
14	German shepherd	1	♂	Healthy

**Table 2:** Detection of periodontal pathogens in dental plaques of individual dogs.

Periodontal Disease Group					Healthy Group				
Dog	*P. g.*	*T. f.*	*T. d.*	*T. p.*	Dog	*P. g.*	*T. f.*	*T. d.*	*T. p.*
**1**	+	+			**8**	+	+		+
**2**	+	+		+	**9**		+		
**3**	+	+		+	**10**	+	+		+
**4**	+	+			**11**	+	+		+
**5**	+	+	+		**12**	+	+		+
**6**	+	+		+	**13**		+		
**7**	+	+	+		**14**	+	+		

*P. g*.—*Porphyromonas gulae*, *T. f.*—*Tannerella forsythia*, *T. d.*—*Treponema denticola*, and *T. p.*—*Treponema putidum*.

**Table 3:** Primer sequences and PCR conditions for the detection of periodontal pathogens.

Species (Gene)	Primer Sequence (5′ to 3′)	PCR Conditions	Length (bp)	Source
*Porphyromonas gulae* (fragment of 16S rRNA gene)	TTGGTTGCATGATCGGG	94 °C 5 min, 35× [94 °C 30 s, 58 °C 1 min, 72 °C 30 s] 72 °C 5 min	300	[21]
	GCTTATTCTTACGGTACATTCAYA			
*Tannerella forsythia* (fragment of 16S rRNA gene)	GCGTATGTAACCTGCCCGCA	95 °C 2 min, 36× [95 °C 30 s, 60 °C 1 min, 72 °C 1 min] 72 °C 2 min	641	[35]
	TGCTTCAGTGTCAGTTATACCT			
*Treponema denticola* (*flaB2* gene)	ACGGYATTTCYTTTATTCAAGTTGC	94 °C 5 min, 45× [94 °C 30 s, 63 °C 30 s, 72 °C 40 s] 72 °C 5 min	471	[27]
	CGAGTCTGTTYTGGTATGCACC			

## Data Availability

Not applicable.
